# Rapid maxillary expansion in pediatric patients with obstructive sleep apnea: an umbrella review

**DOI:** 10.1016/j.bjorl.2023.02.004

**Published:** 2023-02-17

**Authors:** Denise Fernandes Barbosa, Laura Fernandes Bana, Maria Cristina Buta Michel, Miguel Meira e Cruz, Edilson Zancanella, Almiro José Machado Júnior

**Affiliations:** a Universidade de Campinas (UNICAMP), Departamento de Otorrinolaringologia e Cirurgia de Cabeça e Pescoço, Campinas, SP, Brazil; b Universidade de Campinas (FOP-UNICAMP), Faculdade de Odontologia, Departamento de Morfologia, Piracicaba, SP, Brazil; c Universidade de Araras, Fundação Hermínio Ometto, Araras, SP, Brazil; d Centro Cardiovascular da Universidade de Lisboa, Faculdade de Medicina da Universidade de Lisboa, Unidade de Sono, Lisboa, Portugal

**Keywords:** Rapid maxillary expansion, Obstructive sleep apnea, Children, Systematic review, Meta-analysis

## Abstract

**Objective:**

To compare polysomnographic parameters with others from the literature in order to provide more accurate information about Rapid Maxillary Expansion (RME) for treating Obstructive Sleep Apnea (OSA) in children, through raising the question: Is RME a good option for treating OSA in children? Prevention of mouth breathing during children's growth remains a challenge with significant clinical consequences. In addition, OSA induces anatomofunctional changes during the critical period of craniofacial growth and development.

**Methods:**

The Medline, PubMed, EMBASE, CINAHL, Web of Science, SciELO and Scopus electronic databases were searched up to February 2021 for systematic reviews with meta-analysis in the English language. Among 40 studies on RME for treating OSA in children, we selected seven in which polysomnographic measurements of the Apnea-Hypopnea Index (AHI) had been made. Data were extracted and examined in order to clarify whether any consistent evidence exists for indicating RME as a treatment for OSA in children.

**Results:**

We found no consistent evidence favoring RME for long-term treatment of OSA in children. All the studies presented considerable heterogeneity due to variability of age and length of follow-up.

**Conclusion:**

Through this umbrella review, the need for methodologically better studies on RME is supported. Moreover, it can be considered that RME is not recommended for treating OSA in children. Further studies and more evidence identifying early signs of OSA are necessary in order to achieve consistent healthcare practice.

## Highlights

• The studies of rapid maxillary expansion in obstructive sleep apnea children's treatment are based on low-quality evidence.
• Management decisions should be linked to the phenotype, considering outcomes beyond the apnea-hypopnea index.
• A health policy is needed focusing on respiratory disorders prevention.

## Introduction

Obstructive Sleep Apnea (OSA) is a complex and heterogeneous disorder^1^ characterized by episodes of complete or partial upper airway obstruction or sleep-related breathing disorder consisting of snoring, and by episodes of increased secondary respiratory effort, upper airway resistance and pharyngeal collapsibility during sleep, often resulting in gas exchange abnormalities and sleep disruption.^2^, ^3^, ^4^, ^5^ This condition is present in 2%–5% of children and can occur at any age.^2^, ^3^ It may be the most common sleep disorder.^6^ OSA in children is a severe disease involving diminished quality of life in many aspects, such as neurocognitive and neuropsychomotor impairment, cardiovascular function implications and systemic diseases.^5^, ^6^, ^7^, ^8^, ^9^, ^10^, ^11^ This disorder affects children during critical brain development and craniofacial growth.^10^, ^12^ Genetic influences and environmental stimuli can contribute to facial growth and neuromuscular compensation activity, in order to maintain upper airway patency.^5^

Because of the complexity of OSA, a multidisciplinary healthcare team is required for better results from treatment to be obtained.^6^ Preventing OSA in children is still a challenge with regard to both multidisciplinary team attention and healthcare and evidence-informed decision-making. Mouth breathing is one of the foremost clinical manifestations of OSA, and is accompanied by chronic snoring, increased respiratory effort and arousal,^5^, ^13^, ^14^, ^15^ arising from anatomical and functional imbalance.^5^, ^7^, ^14^, ^15^, ^16^, ^17^, ^18^

Physiological respiratory function is one of the essential stomatognathic functions that require complex interactions of the central and peripheral nervous systems with the respiratory system.^19^ In neonates, respiratory control is relatively immature.^20^ The respiratory reflex is an innate reflex that depends on the level of maturation and function of different neuromuscular structures, which become established through physiological processes. The act of breastfeeding establishes this reflex, which also involves other stomatognathic functions such as sucking and swallowing.^18^ These functions are essential for the growth and development of craniofacial structures in the first years of a child's life.^21^

The number of episodes of obstructive apnea and hypopnea per hour of sleep, as assessed through the Apnea-Hypopnea Index (AHI) indicates the severity of OSA. Most laboratories define OSA in children as follows: mild, when in the range AHI > 1.5 (or AHI > 1–5; moderate, AHI > 5–10; or severe, AHI > 10.^22^, ^23^

Early diagnosis and treatment of OSA may decrease morbidity; however, among children, this is frequently delayed.^12^ Polysomnographic studies need to form part of the screening, diagnosis and follow-up strategies because of the differences in characteristics between adult and pediatric OSA.^12^ Additionally, oximetry is one of the tools most used for preliminary evaluation and provides an abbreviated means for diagnosing OSA.^23^ The cross-culturally validated sleep disorders questionnaire is another sleep assessment tool for initial assessment of OSA children.^24^, ^25^, ^26^ The questionnaire is considered easy to use, is low-cost and is self-administered. Furthermore, some indexes such as the Baby ROMA index,^27^ which is needed for orthodontic screening among children from 2 to 6 years old, take into consideration systemic, skeletal, dental, and functional problems.

The pathophysiology of OSA in children is multifactorial and is divided into factors relating to the associated possibilities for craniofacial development in the upper airway.^23^ Narrowing of the upper airway and presence of neuromuscular disorders increase the risk of craniofacial abnormalities in children with OSA, and certain genetic conditions relating to structural elements lead to disharmony in craniofacial growth and development.^6^, ^7^, ^8^, ^9^, ^28^, ^29^

Diagnosing and treating this breathing disorder in early life are possible.^6^, ^7^, ^9^, ^10^^,^^12^ Moreover, early treatment is deemed necessary for prevention of harmful consequences, even though only a few studies have matched OSA with prevention in this population.^6^, ^8^, ^30^

There are various therapies for OSA^23^, ^31^, ^32^, ^33^ , including adenotonsillectomy as the first-line treatment, with use of positive airway pressure devices, use of nasal devices, myofunctional therapy, sleep surgery and use of oral appliances. Regarding oral appliance therapy, studies on orthodontic/facial orthopedic treatment have provided support for use of the Rapid Maxillary Expansion (RME) technique before midline fusion of the maxilla occurs. RME is an effective treatment for dental crowding and malocclusion in situations of a high arched or narrow hard palate, which is related to presence of OSA in children.^7^, ^8^, ^9^, ^10^, ^23^, ^28^, ^33^^,^^34^

The aims of this umbrella review were the following: 1) To provide a summary of existing research syntheses on RME interventions among children with OSA through evaluation of polysomnographic measurements, especially the Apnea-Hypopnea Index (AHI); and 2) To highlight future research necessities.

## Methods

### Development

Through this study, it was sought to evaluate the effectiveness of RME as a treatment option for OSA, by compiling evidence from multiple research syntheses with polysomnographic measurements, including AHI and other outcomes. We conducted the search strategy in February 2022 using the Patient-Intervention-Comparison-Outcome (PICO) strategy (Table 1). We included relevant studies through using a rigorous electronic search for the terms RME, OSA, AHI, children, systematic review, and meta-analysis.

**Table 1:** Framework for elaborating the PICO strategy.

Population	Children (age from 0 to 18 years) with obstructive sleep apnea.
Intervention	Rapid maxillary expansion.
Comparative group	Apnea hypopnea index, oxygen desaturation index, arousal index.
Outcomes	Improvement of polysomnographic measurements.

### Inclusion criteria

We included all systematic reviews with meta-analysis that assessed OSA in children aged 0–18 years, without gender restriction, who were treated with RME and for whom diagnoses were made using polysomnographic parameters, especially AHI; and for whom pre- and post-treatment data and follow-up evidence were available.

### Exclusion criteria

We established the language restriction of exclusively considering studies in English and excluded theoretical studies and opinions about the primary source of evidence.

### Search strategy

**Figure 1:**
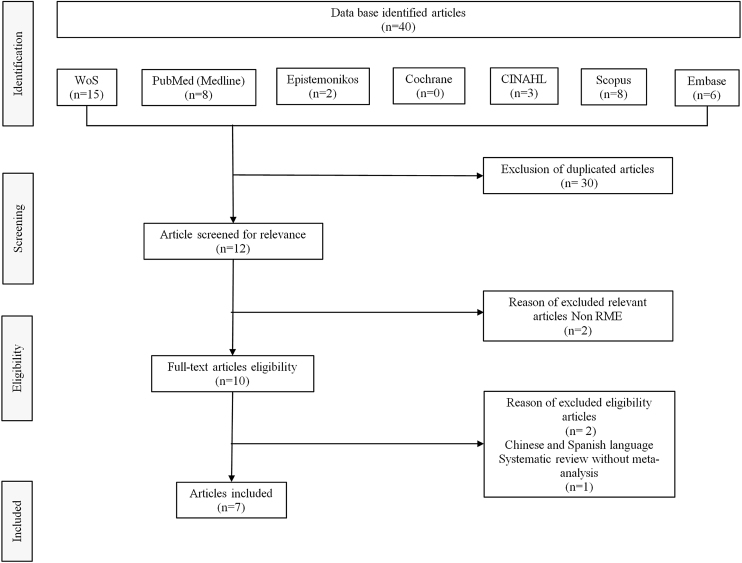
Flow diagram for study selection (Feb 2022).

An electronic database search to identify potentially relevant studies in the Web of Science, PubMed, Scopus, Embase, Cochrane, Epistemonikos, CINAHL and SciELO was conducted in February 2022. Boolean operators (“OR” and “AND”) were used to link search terms based on the PICO strategy. The English-language MeSH research terms used were the following: sleep-disordered breathing, obstructive sleep apnea, RME, children, pediatric, systematic review, and meta-analysis. Out of 40 systematic reviews with meta-analysis on use of RME for treating OSA in children, we selected eight studies on RME in children with OSA in which polysomnographic measurements including AHI were made. However, we then excluded one of these systematic reviews because it did not have a meta-analysis. The flow diagram for study selection is shown in Fig. 1.

### Methodological quality

Initially, we analyzed the polysomnographic parameter outcomes from seven reviews on RME for their similarities and differences and applied a quality assessment. All the information collected is shown in Table 2 (Joanna Briggs Institute Reviewers' Manual 2014). We discussed the qualitative evaluations of the articles retrieved for this study, to produce a consensus.

**Table 2:** Critical appraisal checklist for systematic reviews and research synthesis (Joanna Briggs Institute Reviewers’ Manual 2014).

Study; year	Study site	Outcomes Analysed	Quality assessment^a^										
			1	2	3	4	5	6	7	8	9	10	11
Huynh NT et al.; 2015	Canada	AHI	M	M	M	M	M	M	M	M	M	NM	U
Machado-Júnior AJ et al.; 2016	Brazil	AHI; apnea index; SaO_2_	U	M	M	M	M	NM	NM	M	NM	M	M
Camacho M et al.; 2017	USA	AHI; LSAT	M	M	M	M	M	M	M	M	M	M	M
Vale F et al.; 2017	Portugal	AHI; SaO_2_; AI; REM; SE	M	M	M	M	M	M	M	M	M	NM	U
Sánchez-Súcar AM et al.; 2019	Switzerland	AHI; RDI; SaO_2_, ODI	M	M	M	M	M	M	M	M	M	NM	U
Lin SY et al.; 2020	Taiwan	AHI; SaO_2_; ODI	M	M	M	M	M	M	M	M	M	M	M
Quinzi Vi et al.; 2020	Spain/Canada	AHI; SaO_2_ in the short ‒ and long-term follow-up	M	M	M	M	M	M	M	M	NM	NM	M

OSA, Obstructive Sleep Apnea; AHI, Apnea-Hypopnea Index; SaO_2_, Oxygen Saturation Level; LSAT, Lowest Oxygen Saturation; AI, Arousal Index; UA, Upper Airway; SQ, Sleep Quality; REM, Rapid Eye Movement; SE, Sleep Efficiency; RDI, Respiratory Disturbance Index; ODI, Oxygen Desaturation Index; TST, Total Sleep Time.

M, ‘Met’; NM, ‘Not Met’; U, ‘Unclear’ or NA, ‘Not Applicable’.

a Quality assessment of Joanna Brings Institute for Reviewers’ Manual 2014: 1) Is the review question clarity and explicit stated? 2) Were the inclusion criteria appropriate for the review question? 3) Was the search strategy appropriate? 4) Were the sources and resources used to search for studies adequate? 5) Were the criteria for appraising studies appropriate? 6) Was critical appraisal conducted by two or more reviewers independently? 7) Were there methods to minimize errors in data extraction? 8) Were the methods used to combine studies appropriate? 9) Was the likelihood of publication bias assessed? 10) Were recommendations for policy and/or practice supported by the reported data? 11) Were the specific directives for new research appropriate?

Systematically and independently, two reviewers (B, DF; and B, LF) conducted assessments and manually documented them with regard to each respective database: author, year of publication, title, study design, number of patients, age, methods, outcomes, results and conclusion. The two reviewers discussed their evaluations on qualitative articles in order to develop a consensus. In addition, a third reviewer (M-J, A-J) was consulted in order to validate and control the data in any event of disagreement. The two reviewers undertook several rounds of rereading each review and also searched through the bibliographies, to look for other studies that might not have been found in the initial search.

We summarized these characteristics and findings into a single question: Is RME an effective intervention for controlling the AHI in children with OSA? We sought to communicate all the evidence found through the present review to the multidisciplinary team that cares for children with OSA, so as to guide the team regarding good clinical-practice decision-making.

## Results

Out of 40 systematic reviews with meta-analysis on the use of RME for treating OSA in children, we selected eight studies on RME in children with OSA in which polysomnographic measurements including AHI were made. However, we then excluded one of these systematic reviews because it did not have a meta-analysis. The flow diagram for study selection is shown in Fig. 1.

Initially, we analyzed the polysomnographic parameter outcomes from seven reviews on RME for their similarities and differences and applied a quality assessment. All the information collected is shown in Table 2 (Joanna Briggs Institute Reviewers' Manual 2014). We discussed the qualitative evaluations of the articles retrieved for this study, to produce a consensus. We used the common AHI outcomes analysis in all studies.

The overall results from this review are described in Table 3. All the studies presented considerable heterogeneity in their results from RME interventions among children with OSA. Despite the significant variability observed in this umbrella review, in which the coefficient of variation across the studies was more than 90% (I^2^ > 90%) and which is explained by the nature of AHI measurements expressed in a personalized manner, the evidence of differences between pre- and post-treatment conditions had high significance (p < 0.01). The exception to this was the study by Lin et al.^23^, which had a coefficient of less than 50%, thus indicating that there are some studies that enable selection of experiments with greater similarity.

**Table 3:** AHI findings from systematic reviews with meta-analysis on RME among children with OSA: an umbrella review.

Author; year	Number of studies/children	Age range/mean	AHI findings of pre- and post-treatment RME	Follow-up	Heterogeneity		p-value
					Variation across studies	Level	
Huynh NT et al.; 2015	5 studies/183	18y or younger	MD IV, Fixed, 95% CI 6.19 (5.81, 6.57)	4 weeks, 4–6 to 18 months	98%	High	<0.00001
Machado-Júnior AJ et al.; 2016	10 studies/350	0‒12y/6.7y	MD IV, Fixed, 95% CI −6.86 (−7.18, −6.54)	3 months to 14 years	98%	High	<0.00001
Camacho M et al.; 2017	17 studies/314	7.6 ± 2.0y	MD IV, Random, 95% CI −4.84 (−8.47, −1.21)	Less than 3y	99%	High	<0.0001
			St. Mean Difference IV, Random, 95% CI −1.54 (−2.29, −0.78)	More than 3y	94%	High	<0.00001
Vale F et al.; 2017	5 studies/137	18y or younger	Random, SMD Effect Size, 95% CI 3.24 (0.34, 6.15) (AHI improvement)	12 months	98.02%	High	<0.0001
			Random, SMD Effect Size, 95% CI −2.91 (−4.80, −1.02) (AHI normalization)	12 months to normal	95.53%	High	<0.0001
Sánchez-Súcar AM et al.; 2019	9 studies/283	around 8y	MD, Fixed/Random, 95% CI −6.617 (−6.910, −6.324)/−5.797 (−9.06, −2.5)	Not specified	98.9%	High	=0.000
Lin SY et al.; 2020	14 studies/1064	under 19y/6.5 ± 0.2y	MD, Effect Size, 95% CI −1.90 (−5.33, 1.53)	3-months	44.9%	High	<0.001
Quinzi, VI et al.; 2020	6/102	6.7 ± 1.3y	MD IV, Random, 95% CI 5.11 (4.58, 5.64)	≤3 year in 79 children	97%	High	<0.00001
				>3 years in 23 children			

AHI, Apnea-Hypopnea Index; RME, Rapid Maxillary Expansion; OSA, Obstructive Sleep Apnea; MD, Mean Difference; y, years old; IV, Inverse Variable; I_2_, Percentage of variation across studies; NS, Not Specified.

Our synthesis of findings from systematic reviews with meta-analysis on outcomes from RME for AHI control among children with OSA, in Table 4, shows treatments and recommendations for healthcare and evidence-informed decision-making and future research. Instead of the high heterogeneity with very low quality of studies regarding RME, the outcomes relating to AHI control shown in the study by Quinzi et al.^9^ indicated that RME was effective over the long term. Moreover, the study by Vale et al.^35^ indicated that RME was an appropriate alternative for treating craniofacial abnormalities; whereas the study by Lin et al.^23^ showed that RME may not be effective. On the other hand, all the others studies , by Machado et al.^6^, Huynh et al.^7^, Sánchez-Súcar et al.^14^ and Camacho et al.^33^, demonstrated that RME might be effective. Quinzi et al.^9^ recommended investigation of RME efficacy in long-term treatment of OSAS and highlighted the importance of AT combined with RME treatment. Vale et al.^35^ indicated RME as an auxiliary method for treating children with OSAS risk factors such as craniofacial abnormalities. Machado et al.^6^ suggested assessing whether the efficacy of this treatment was retained throughout adulthood. Huynh et al.^7^ concluded that the quantity and quality of published papers could be improved if the study design envisaged larger sample sizes and specific inclusion and exclusion criteria. Sánchez-Súcar et al.^14^ pointed out the limitations of the various methods used and the publication bias, and the lack of high-quality randomized case/control studies. Although Camacho et al.^33^ confirmed the effects of RME with regard to reducing and normalizing the AHI values, they pointed out the lack of quantity and quality of studies assessing the efficacy of RME for treating OSA in children. In addition, they suggested that the Consolidated Standards of Reporting Trials should be used to guide the research design.

**Table 4:** Synthesis of findings from systematic reviews with meta-analysis on outcomes from RME for AHI control among children with OSA.

Author; year	Findings of RME outcome of OSA treatment	The necessity of RME investigation
Huynh NT et al.; 2015	May be effective.	More studies with larger sample sizes and with speciﬁc inclusion and exclusion criteria.
Machado-Júnior AJ et al.; 2016	May be effective.	More studies with follow-up.
Camacho M et al.; 2017	May be effective in the short term (<3-year follow-up).	More studies with long-term data (3-years of follow-up) to determine the growth effect and spontaneous OSA resolution.
Vale F et al.; 2017	An appropriate alternative in craniofacial abnormalities.	More studies with CONSORT guidelines.
Sánchez-Súcar AM et al.; 2019	May be effective in mild to moderate AHI, and effective in severe AHI with A&T.	More studies with measurement protocols for review comparison.
Lin SY et al.; 2019	It may not be effective in reducing AHI.	Trials that evaluate the newer technologies or combination therapies to identify the best treatment for pediatric OSA
Quinzi, Vi et al.; 2020	RME has efficacy in OSA.	Studies with OSAS treatment long-term.

RME, Rapid Maxillary Expansion; AHI, Apnea Hypopnea Index; OSA, Obstructive Sleep Apnea; CONSORT, Consolidated Standards of Reporting Trials; A&T, Adenotonsillectomy.

## Discussion

Due to the quality of the studies included and the significant heterogeneity among them, we were unable to reach conclusions from this umbrella study that would be similar to those of most of the systematic review studies with meta-analysis that were evaluated. Five out of seven studies showed that RME may or may not be effective with regard to AHI improvement in children.

While RME is a well-accepted orthopedic procedure for managing structural and functional problems in the midface,^29^ upper airspace improvement and stability are the main long-term issues relating to treatments for OSA among children. In addition, it is premature to speculate about use of RME as a treatment for nasal obstruction, given the significant risk of bias and high heterogeneity of results regarding improvement of OSA, especially with regard to long-term stability.^6^, ^9^, ^33^, ^34^^,^^36^ On the other hand, the effects of RME may be cloaked because maxillary constriction can play a role in the etiology of OSA^36^. At the same time, coadjuvant therapy among children with severe OSA, such as adenotonsillectomy in situations of a narrow maxilla, has been shown to provide improvement of the nasal airway dimensions and airflow.^14^, ^34^, ^37^ This may cloak the effects of RME because maxillary constriction may play a role in the etiology of OSA.^36^ Nevertheless, what we want to highlight is that OSA may negatively affect a child for the rest of their life.^7^, ^36^, ^38^

We compared improvements in AHI achieved through RME interventions that were reported in selected systematic reviews with meta-analysis. We noted that there was a correlation between skeleton-related orofacial dysfunctions and presence of OSA among these children.^7^, ^33^, ^39^, ^40^ Vale et al.^35^ recommended RME for treatment of OSA in children with craniofacial abnormalities. However, orthodontic and craniofacial abnormalities are often neglected in children with OSA.^6^, ^8^ Meanwhile, Huynh et al.^7^ suggested that correcting craniofacial structure imbalances under the optimal conditions afforded by childhood growth may diminish snoring and OSA and would likely improve polysomnographic parameters such as AHI, oxygen saturation index, arousal index, upper airway volume or structures and sleep quality, especially over the short term (<3 years of follow-up). In addition, regarding the follow-up, Camacho et al.,^33^ Machado-Junior et al.^6^ and Quinzi et al.^9^ pointed out that there is a need for more long-term studies (>3 years of follow-up) and for more randomized clinical trials with long-term follow-up, in order to assess whether the effectiveness of this treatment is maintained throughout adulthood.^6^

Huynh et al.^7^, Sánchez-Súcar et al.^14^, Lin et al.,^23^, Camacho et al.,^33^ Vale et al.,^35^ Calvo-Henriquez et al.^37^ and a recent systematic review^41^ came to similar conclusions regarding the heterogeneity of the results observed. They confirmed the importance of instituting standardized trial guidelines for research designs, to reduce bias and improve the inclusion and exclusion criteria. In this context, in future clinical trials, patient selection would likely benefit from including phenotypic approaches and personalized medicine, so as to gain understanding of therapeutic mechanisms and thereby improve diagnoses, prognoses and clinical management.^42^

We found a gap in the literature with regard to treatment plans. It needs to be considered that an adequate treatment plan stemming from early-stage diagnosis helps to identify respiratory disorders, reduce adverse health outcomes^11^, ^38^ and prevent malocclusion.^27^ Treatments should focus on amending craniofacial development, given that there is a direct relationship between malocclusion and other OSA-related orofacial deformities, considering also that there is no robust scientific evidence to reach complete resolution of OSA.^23^

If it is supposed that a direct relationship exists between malocclusion and other OSA-related orofacial deformities, the question of what to do regarding treatments that do not correct craniofacial development arises. These treatment may include adenotonsillectomy, CPAP and other ineffective therapies.^6^, ^10^ From the systematic reviews with accurate meta-analysis that we selected, there was no robust scientific evidence to support treatment of OSA patients with RME, surgically assisted RME or maxillomandibular surgical advancement.^30^, ^41^

Trials that evaluate the latest technologies or combined therapies are also needed in order to identify the best treatment for pediatric OSA, for future networked meta-analysis.^23^ For this reason, we understand why there is discordance between the American Academy of Pediatrics^43^ and the European Respiratory Society^4^ with regard to recommending RME as a treatment for OSA in children. Importantly, our comprehensive survey showed that there is insufficient evidence of effectiveness regarding RME treatment.^23^, ^41^, ^44^ Thus, other challenges and perspectives regarding prognoses and optimal treatment among children with OSA need to be considered.^45^ These may include patient history and clinical sleep records,^24^ nocturnal pulse oximetry,^23^ OSA questionnaires^25^, ^26^ and phenotypic markers.^42^, ^46^

There is a need for more studies, especially with regard to public preventive healthcare policies for children with OSA. The links connecting breastfeeding action to pediatric sleep-disordered breathing,^47^ craniofacial growth and development in the postnatal period and first years of life^48^, ^49^ need to be considered. The pediatric population under two years of age is a unique subgroup with a predisposition to upper airway obstruction with symptoms during wakefulness and requires age-appropriate interventions.^11^ Moreover, preventive treatment should act at the primary level of prevention, so as to improve anatomical form and systemic function and promote establishment of nasal breathing at the early stage of growth and development. Additionally, new studies should explore gaps in knowledge relating to long-term issues and orthopedic development of the stomatognathic system.

Strategic healthcare and evidence-informed decision-making for preventive action connecting breastfeeding action to pediatric sleep-disordered breathing^47^, craniofacial growth and development in the postnatal period and first years of life^48^, ^49^, need to be considered. This would be preferable to working with installed mouth breathing using methods that do not present any apparent efficacy or effective treatment methods. Regarding the strengths of this review, we recommend that new studies should be conducted to explore the gaps in knowledge found in the literature.

## Conclusion

The conclusion from this umbrella review is that it is premature to speculate that RME forms a treatment for OSA in children. Because of the low quality of evidence and high heterogeneity between studies, we believe that RME treatment should not be recommended for children with OSA. Clinical trial guidelines are needed in order to improve quality, avoid heterogeneity among studies and enable better outcomes. Management decisions should be linked to underlying phenotypes and consider outcomes other than the AHI. Future strategic campaigns are needed to raise awareness among healthcare and evidence-informed decision-making regarding the best practices in relation to prevention of OSA among children. In addition, more evidence to make it possible to establish healthcare policies focusing on primary prevention of respiratory disorders should be obtained.

## Future directions

Future long-term prospective research should prioritize methodological quality, so as to avoid selection bias through sample homogeneity, in terms of both patient age and length of treatment, with timely therapy. Overall, the present review indicated that preventive action to reestablish nasal breathing in the pediatric population is needed in order to avoid deviation from normal growth and development. In addition, the AHI and variables relating to clinical characteristics should be considered, including risk factors such as nasal obstruction and mouth breathing, anatomical and functional changes, craniofacial abnormalities, quality of life and cognitive and behavioral factors.

## Funding

The authors received no financial support for this article's research, authorship, or publication.

## Conflicts of interest

The authors declare no conflicts of interest.
